# Prevalence of traditional cardiovascular risk factors for coronary artery disease and elevated fibrinogen among active military personnel in Republic of Serbia: A cross-sectional study

**DOI:** 10.5937/jomb0-33428

**Published:** 2022-04-08

**Authors:** Milena Pandrc, Nenad Ratković, Vitomir Perić, Maja Stojanović, Vanja Kostovski, Nemanja Rančić

**Affiliations:** 1 Military Medical Academy, Clinic for Urgent Internal Medicine, Belgrade; 2 University of Defence in Belgrade, Military Medical Academy, Medical Faculty, Belgrade; 3 Military Medical Academy, Sector for treatment, Belgrade; 4 Military Medical Centre Karaburma, Belgrade; 5 University of Defence, Military Medical Academy, Clinic for Cardiothoracic Surgery, Belgrade; 6 Military Medical Academy, Center for Clinical Pharmacology, Belgrade

**Keywords:** traditional cardiovascular risk factors, coronary artery disease, fibrinogen, active military personnel, tradicionalni kardiovaskularni faktori rizika, koronarna arterijska bolest, fibrinogen, aktivna vojna lica

## Abstract

**Background:**

It is well known that less than 1% of the population achieves ideal cardiovascular health, and 65% of patients do not have their conventional risk biomarkers under control. Military service has its own particularities that may contribute to cardiovascular risk.

**Methods:**

To define the preventive strategy goals, we analysed the prevalence of traditional cardiovascular risk factors for coronary artery disease and elevated fibrinogen among active military personnel in the Republic of Serbia.

**Results:**

The cross-sectional study included 738 individuals older than 20 years, mostly between 31 and 40 years old. The mean value of SBP for the whole group was 122.39± 9.42 mmHg, and for the DBP, it was 79.94±6.56 mmHg. Among active military personnel, 72.7% (533) had prehypertension, and 13.8% (101) was hypertensive. Both body mass and BMI index among the observed age subgroups were found to increase with the age of the patients and cholesterol values. HDL cholesterol values also differed statistically significantly between age subgroups, with the proportion of individuals with HDL less than 1.5 mmol/L in all subgroups being about 85%, the only in the 41-50 age group was lower, 76.4%. LDL cholesterol and the proportion of individuals who had LDL 3.5 increases with the age of patients, and an identical trend was recorded with triglycerides. With ageing, fibrinogen levels increased.

**Conclusions:**

Those findings considering cardio and cerebrovascular risk factors would help create a new approach for primary prevention for these categories of individuals.

## Abstract (sr)

**Uvod:**

Dobro je poznato da manje od 1% populacije ima idealno kardiovaskularno zdravlje, kao i da 65% pacijenata nema kontrolu nad konvencionalnim biomarkerima rizika. Vojna služba ima svoje osobitosti koje mogu doprineti kardiovaskularnom riziku.

**Metode:**

Da bismo definisali ciljeve preventivne strategije, analizirali smo prevalenciju tradicionalnih kardiovaskularnih faktora rizika za koronarnu arterijsku bolest i povišenog fibrinogena među aktivnim vojnim licima vojske Republike Srbije.

**Rezultati:**

Studija preseka je obuhvatila 738 osoba starijih od 20 godina, uglavnom između 31 i 40 godina. Srednja vrednost SBP-a za celu grupu bila je 122,39 ± 9,42 mmHg, a za DBP 79,94 ± 6,56 mmHg. Među ispitivanom populacijom 72.7% (533) je imalo prehipertenziju, a 13,8% (101) hipertenziju. Utvrđeno je da se i telesna masa i indeks telesne mase među posmatranim starosnim podgrupama povećavaju sa godinama pacijenata, kao i vrednosti ukupnog holesterola. Vrednosti HDL holesterola su se takođe statistički značajno razlikovale među starosnim podgrupama, pri čemu je udeo pojedinaca sa HDL-om manjim od 1,5 mmol/L u svim podgrupama bio oko 85%, dok je jedino u starosnoj grupi od 41-50 godina bio niži, 76,4%. LDL holesterol, kao i udeo pojedinaca koji su imali LDL ³ 3,5, raste sa starenjem pacijenata, a identičan trend je zabeležen i kod triglicerida. Sa starenjem, nivo fibrinogena se povećava.

**Zaključak:**

Dobijeni rezultati koji se odnose na kardio i cerebrovaskularne faktore rizika bi mogli doprineti stvaranju novog pristupa u primarnoj prevenciji kod ovih kategorija bolesnika.

## Introduction

It is well known that less than 1% of the population achieves ideal cardiovascular health, and 65% of patients do not have their conventional risk biomarkers under control [1] [2]. Although recent data are very suggestive for primary prevention in people with multiple risk factors, fewer than 10% of those individuals have all of them adequately controlled [2] [3] [4] [5]. The primary prevention strategy in individuals with multiple risk factors is based on the fact that the first atherosclerotic changes-fat spots and stripes, consisting mainly of macrophages filled with LDL cholesterol, appear early in life, even in childhood [6] [7]. These findings underline the need for lipid status screening to provide better objectivity in assessing cardiovascular risk and the rationale for the early introduction of lifestyle changes and drug therapy. Except for lipid status, cardiovascular risk evaluation includes fibrinogen levels, a widely used surrogate cardiovascular marker, which also has a predictive value [8]. Halle et al. [9] underlined a clear link between higher normal fibrinogen and the expression of a more atherogenic LDL subfraction phenotype independent of body mass index, age, other serum lipids, and insulin resistance in a healthy person nonsmoking male. A meta-analysis with about 4000 coronary heart disease cases indicated that an increase in plasma fibrinogen level per 1 g/L was followed by a relative risk ratio increment of 1.8 [10]. A recent meta-analysis with 246,669 otherwise healthy participants underlined the clear benefit of assessing the CRP or fibrinogen level in individuals at intermediate risk for a cardiovascular event considering prevention of an additional event over a period of 10-year follow-up [11]. These findings reinforce the evidence that fibrinogen should be estimated in coronary risk assessment.

Considering the role of lipid disorders in atherosclerosis, it is important to have a screening program for the early detection of lipid disorders. Active military personnel selection should be based on »a kind of healthy worker effect« or »Healthy Warrior Effect« to provide the population that is healthier than the general one [12] [13]. Military service has its own particularities that may contribute to cardiovascular risk [14] [15] [16]. Previous referred findings are the basis for the scientific project »Primary prevention of ischemic heart disease among active military personnel and civilian personnel in the military in the Republic of Serbia«, which aims to implement actual prevention recommendations among active-duty military personnel and military personnel. This part of the population is under systemic control and the possibility of daily health status checks. This is objectified by general medical examination yearly (younger than 40 years) or every second year (older than 40 years).

The aim of the study is to point out the prevalence of hyperlipidemia and elevated fibrinogen among active-duty military personnel in the Republic of Serbia.

## Methods

### Type of the study and participants

This is a cross-sectional study (2018-2019), included a sample of 738 active military personnel (20+ years) of Serbia. The study was conducted in the Military Medical Academy and Military Medical Centre Karaburma. All procedures performed in the study involving human participants were in accordance with the ethical standards of the Ethical Commission of Belgrade University of Defence. The study population included 738 males, divided into two groups. The first one consists of 289 individuals younger than 40 years, and then the second one includes 489 people older than 40.

### Some epidemiological and anthropometric Characteristics were checked to assess cardio -metabolic risk

Anthropometric measurements and calculationsincluded body weight and height, as well as bodymass index (BMI, calculated as weight (kg)/squared body height (m^2^)). Recognised criteria were used forthe assessment of overweight and obesity versus normal BMI. Cutoff value for overweight and obesity was BMI ≥ 25 kg/m^2^. The systolic and diastolic blood pressure (SBP and DBP) cardiologists measured by the traditional sphygmomanometer with a participant in a sitting position. The values of systolic and diastolic blood pressure were recorded as the arithmetic mean of three repeated measurements. In preparation for measurements, the participants were seated and rested quietly for at least five minutes before taking the first BP measurement. The right arm was used for all blood pressure measurements. All participants had BP measurements always taken by the same researcher and with the same-sized cuff for adults. Time intervals between the measurements were 5-10 minutes. The first (for systolic) and fifth (for diastolic) Korotkoff sounds were recorded for each of the 3 measurements [4] [15].

According to WHO, the examinees without antihypertensive therapy over the last 4 months were classified according to values of blood pressure into the following categories: normal blood pressure (systolic blood pressure - SBP and diastolic blood pressure - DBP: <120 and <80 mmHg); prehypertension (SBP/DBP: 120-139 and/or 80-89 mmHg); and hypertension (SBP/DBP: ≥140 and/or ≥90 mmHg). All individuals who used antihypertensive therapy over the previous 4 weeks were included in the category of hypertensive individuals [5] [17].

### Biochemical data analysis

By using the Auto Analyzer HITACHI 7020 (902), Japan, the following biochemical analyses were done: high-density lipoprotein cholesterol (HDL), triglycerides (Tg), total cholesterol (TC). In addition, low-density lipoprotein cholesterol (LDL) was calculated by the Friedewald formula (LDL=TC-HDL-TG/2.2) [18]. Fibrinogen was done by analysers using kits from Dade Behring Marburg GmbH.

### Statistical analysis

The data were analysed using the Statistical Package for the Social Sciences IBM-SPSS, version 26.0. Categorical variables were presented as frequency and were analysed using the Chi-square test. All continuous variables are presented as median (interquartile range: 25-75th percentile) or mean±standard deviation for the data that are not normally or normally distributed, respectively. The Shapiro-Wilk test was used to test the normality of data distribution. For intergroup comparisons, the Kruskal-Wallis test for non-parametric variables and ANOVA for parametric variables was used. Spearmen's coefficient correlation tested the relationship between variables. Also, the relationship between the fibrinogen as a dependent variable and other variables were examined using multiple linear regression analysis. Statistical significance was defined as p<0.05 for all comparisons.

## Results

**Figure 1:**
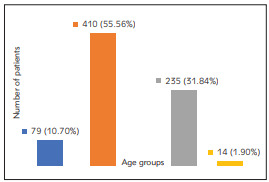
Distribution of the participants according to the age

The cross-sectional study included 738 individuals older than 20 years. The median age of the participants was 38 years for the whole group. Most of the group consisted of individuals between 31 and 40 years old Figure 1.

The mean value of SBP for the whole group was 122.39±9.42 mmHg, and for the DBP, 79.94±6.56 mmHg. Among active military personnel, 72.7% (533) had prehypertension, and 13.8% (101) was hypertensive.

A statistically significant difference was found in the body mass and BMI index among the observed age subgroups. Both variables gradually increased with the age of the patients, so that the highest average values were in the age group of 51-60 years; those participants had approximately body mass of 97 kg and BMI almost 29.5 kg/m^2^. The proportion of patients with BMI ≥25 kg/m^2^ grew with age, so in the youngest group, there were only 58.2% patients with BMI ≥25 kg/m^2^, while in the group older than 51 years, there were 100% patients. Both systolic and diastolic pressure are statistically the highest in the oldest group and increase gradually with age. The proportion of hypertensive patients also increased with age; in the youngest group, there were only 6.4% of patients with hypertension, and in the oldest group, 21.4%. Cholesterol values also increased with age; statistically, significantly higher cholesterol levels were recorded in the older group compared to the previous three subgroups. It was similar with cholesterol ≥5.2 mmol/L; the youngest group had a frequency of 36.7% and the oldest 78.6%. HDL cholesterol values also differed statistically significantly between age subgroups, with the proportion of individuals with HDL less than 1.5 mmol/L in all subgroups being about 85%, the only in the 41-50 age group was lower, 76.4%. LDL cholesterol and the proportion of individuals who had LDL ≥3.5 increased with the age of patients, and an identical trend was recorded with triglycerides (Table 1).

**Table 1:** Distribution of clinical and biochemical characteristics among active-duty military personnel according to the agegroups * – Kruskal-Wallis test; ** – Chi-square test; # – ANOVA; TCH, Cholesterol; Tg – Triglyceride; BMI – Body Mass Index; IQR– interquartile range, MV – mean value, SD – standard deviation; BP – Blood pressure; SBP – Systolic blood pressure; DBP – Diastolic blood pressure; Normal BP (SBP<120 mmHg and DBP<80 mmHg); Prehypertension (SBP=120–139 mmHg and/or DBP=80–89 mmHg); Hypertension (SBP≥140 mmHg and/or DBP≥90 mmHg, or current treatment with antihypertensive medications).

Characteristics	Age groups; median (IQR), number (%) or MV±SD				
	20–30 years<br>(n=79)	31–40 years<br>(n=410)	41–50 years<br>(n = 235)	51–60 years<br>(n=14)	p value
Body mass, kg	85.00<br>(80.00–90.00)	88.00<br>(80.00–96.12)	90.40<br>(83.17–97.50)	96.90<br>(89.65–106.62)	<0.001^*^
Body height, cm	183.00<br>(177.00–186.00)	181.00<br>(177.00–186.00)	181.00<br>(177.00–186.00)	181.50<br>(174.50–185.50)	0.524^*^
BMI, kg/m^2^	25.08<br>(24.43–27.00)	26.81<br>(24.80–28.81)	27.20<br>(25.60–29.50)	29.49<br>(28.47–31.43)	<0.001^*^
<24.99	33 (41.8%)	104 (25.6%)	44 (18.8%)	-	<0.001^**^
≥25.00	46 (58.2%)	302 (74.4%)	190 (81.2%)	14 (100.0%)	
Systolic blood pressure, mmHg	119.60±7.69	121.43±9.13	124.81±9.94	125.36±8.65	<0.001^#^
Diastolic blood pressure, mmHg	78.72±5.72	79.47±6.62	81.03±6.63	82.50±5.09	0.004^#^
Normal bood pressure	11 (14.1%)	65 (16.0%)	23 (9.8%)	-	<0.001^**^
Prehypertension	63 (79.5%)	303 (74.5%)	157 (67.1%)	11 (78.6%)	
Hypertension	5 (6.4%)	39 (9.5%)	54 (23.1%)	3 (21.4%)	
Total cholesterol, mmol/L	4.87 (4.20–5.48)	5.10 (4.46–5.88)	5.61 (4.93–6.29)	6.01 (5.44–6.34)	<0.001^*^
<5.2 mmol/L	50 (63.3%)	216 (52.8%)	82 (35.2%)	3 (21.4%)	<0.001^**^
≥5.2 mmol/L	29 (36.7%)	193 (47.2%)	151 (64.8%)	11 (78.6%)	
HDL cholesterol, mmol/L	1.26 (1.10–1.42)	1.18 (0.85–1.67)	1.26(1.12–1.49)	1.28 (1.04–1.48)	0.001^*^
≥1.5 mmol/L	12 (15.2%)	61 (15.0%)	55 (23.6%)	2 (15.4%)	0.033^**^
<1.5 mmol/L	67 (84.8%)	346 (85.0%)	178(76.4%)	11 (84.6%)	
LDL cholesterol, mmol/L	3.01 (2.51–3.61)	3.27 (2.75–4.00)	3.60 (3.02–4.15)	3.71 (3.31–4.21)	<0.001^*^
<3.5 mmol/L	58 (74.4%)	244 (60.4%)	105 (45.7%)	4 (30.8%)	<0.001^**^
≥3.5 mmol/L	20 (25.6%)	160 (39.6%)	125 (54.3%)	9 (69.2%)	
Triglycerides, mmol/L	0.92 (0.74–1.35)	1.18 (0.85–1.67)	1.37 (0.93–1.99)	2.03 (1.09–2.85)	
<1.7 mmol/L	71 (89.9%)	308 (75.5%)	151 (64.8%)	6 (42.9%)	<0.001^*^
≥1.7 mmol/L	8 (10.1%)	100 (24.5%)	82 (35.2%)	8 (57.1%)	<0.001^**^
Fibrinogen, mmol/L	2.30 (2.00–2.50)	2.70 (2.30–3.10)	3.20 (2.80–3.50)	3.30 (2.70–3.80)	<0.001^*^

**Figure 2:**
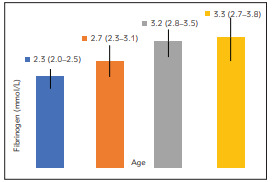
Fibrinogen by the age groups (Fibrinogen values were shown as median with interquartile range: 25–75. percentile)

With ageing, fibrinogen levels increased. Figure 2 showed that the median fibrinogen value increased from 2.30 mmol/L in the youngest group to 3.3 mmol/L in the oldest group. There were statistically significant differences among age groups considering all observed variables of lipid status (total cholesterol, LDL and HDL cholesterols, triglycerides). With ageing, the proportion of the patients with increased lipids' levels grew (Table 2).

**Table 2:** Stratified risk (low, moderate, high) within each fraction of lipid status (total cholesterol, HDL cholesterol, LDL cholesterol, triglycerides)

Characteristics	Age groups; median (IQR) or number (%)				
	20–30 years(n = 79)	31–40 years(n = 410)	41–50 years(n = 235)	51–60 years(n = 14)	p value
Total cholesterol, mmol/L					
Low <5.2	50 (63.3)	216 (52.8)	82 (35.2)	3 (21.4)	<0.001^*^
Moderate 5.2–6.2	26 (32.9)	127 (31.1)	84 (36.1)	6 (42.9)	
High >6.2	3 (3.8)	66 (16.1)	67 (28.8)	5 (35.7)	
LDL cholesterol, mmol/L					
Low <3.5	58 (74.4)	244 (60.4)	105 (45.7)	4 (30.8)	<0.001^*^
Moderate 3.5–4.1	17 (21.8)	71 (17.6)	61 (26.5)	6 (46.2)	
High >4.1	3 (3.8)	89 (22.0)	64 (27.8)	3 (23.1)	
HDL cholesterol, mmol/L					
Low >1.5	12 (15.2)	61 (15.0)	55 (23.6)	2 (15.4)	0.033^*^
Moderate 1.0–1.5	57 (72.2)	273 (67.1)	155 (66.5)	9 (69.2)	
High <1.0	10 (12.7)	73 (17.9)	23 (9.9)	2 (15.4)	
Triglycerides, mmol/L					
Low <1.69	71 (89.9)	308 (75.5)	151 (64.8)	6 (42.9)	<0.001^*^
Moderate 1.7–2.25	4 (5.1)	47 (11.5)	39 (16.7)	3 (21.4)	
High > 2.26	4 (5.1)	53 (13.0)	43 (18.5)	5 (35.7)	

The significant positive correlations among age and all other analysed parameters were recorded. With ageing, all observed parameters grew. The correlation matrix illustrated that age was positively strongly correlated with all observed variables. A mutual correlation was also found between other parameters, so it could be concluded that the values of observed cardiovascular risk factors increased with age. In addition to age, fibrinogen was also seen in astrong positive correlation with cholesterol, LDL cholesterol and triglycerides (Table 3). Also, multiregression analysis was performed and obtained a significant model (F=7.577; p <0.001). The only significant variable that stood out was age. Ageing explains most of the variability of fibrinogen; the fibrinogen grew with increasing age (Table 4).

**Table 3:** The correlation matrix illustrated age was strongly positively correlated with all observed variables r – Spearman’s rho

Variables		Age (years)	TotalCholesterol(mmol/L)	LDL cholesterol(mmol/L)	HDLcholesterol(mmol/L)	Triglycerides(mmol/L)	Fibrinogen(mmlo/L)	Body massindex(kg/m^2^)	Systolicblood pressure (mmHg)	Diastolicblood pressure (mmHg)
Age, years	r	1.000								
	p									
Total cholesterol, mmol/L	r	0.297	1.000							
	p	<0.001								
LDL cholesterol, mmol/L	r	0.221	0.928	0.367	0.027	1.000				
	p	<0.001	<0.001	<0.001	0.473					
HDL cholesterol, mmol/L	r	0.086	0.128	-0.442	1.000					
	p	0.019	0.001	<0.001						
Triglycerides, mmol/L	r	0.206	0.498	1.000						
	p	<0.001	<0.001							
Fibrinogen, mmol/L	r	0.465	0.231	0.137	0.054	0.201	1.000			
	p	<0.001	<0.001	0.001	0.199	<0.001				
Body mass index, kg/m^2^	r	0.219	0.179	0.274	-0.204	0.166	0.139	1.000		
	p	<0.001	<0.001	<0.001	<0.001	<0.001	0.001			
Systolic blood pressure, mmHg	r	0.215	0.143	0.204	-0.068	0.115	0.184	0.288	1.000	
	p	<0.001	<0.001	<0.001	0.067	0.002	<0.001	<0.001		
Diastolic blood pressure, mmHg	r	0.149	0.086	0.142	-0.060	0.059	0.168	0.234	0.599	1.000
	p	<0.001	0.019	<0.001	0.104	0.117	<0.001	<0.001	<0.001	

**Table 4:** Multi-regression analysis with fibrinogen as a dependent variable

Variables	Unstandardised	Coefficients	Sig.	95.0% Confidence Interval for B	
	B	Std. Error		Lower Bound	Upper Bound
Age, years	0.044	0.007	0.000	0.030	0.058
Total cholesterol, mmol/L	0.010	0.099	0.916	-0.184	0.205
LDL cholesterol, mmol/L	0.057	0.106	0.594	-0.152	0.266
HDL cholesterol, mmol/L	0.001	0.007	0.940	-0.014	0.015
Triglycerides, mmol/L	0.018	0.055	0.750	-0.091	0.126
Systolic blood pressure, mmHg	-0.004	0.006	0.519	-0.016	0.008
Diastolic blood pressure, mmHg	0.007	0.008	0.428	-0.010	0.023

According to fibrinogen values, all patients were divided into terciles; one-third of patients from the smallest to the largest fibrinogen value. The higher fibrinogen values significantly increased the patient's age, blood pressure, total cholesterol, LDL cholesterol and triglycerides (Table 5).

**Table 5:** The higher fibrinogen values significantly increased the patient’s age, blood pressure, total cholesterol, LDL cholesterol and triglycerides ^#^ One-Way ANOVA; ^*^ Kruskal-Wallis test

Characteristics	FIBRINOGEN tertiles; median (IQR), number (%) or MV±SD			
	Low (2.0–2.5)	Mid (2.5–3.1)	High (3.1–7.0)	p value
Age, years	36.00 (30.00–38.00)	38.00 (35.00–41.00)	41.00 (37.00–45.00)	<0.001^*^
Body mass, kg	86.00 (80.00–95.00)	90.00 (80.90–96.00)	88.35 (81.52–97.05)	0.159^*^
Body height, cm	183.00 (177.00–186.00)	181.00 (177.50–186.00)	180.50 (176.50–185.00)	0.090^*^
Body mass index, kg/m^2^	26.00 (24.52–28.05)	27.00 (25.00–28.83)	27.00 (25.12–29.41)	0.002^*^
Systolic blood pressure, mmHg	120.30±8.59	122.63±9.13	124.51±10.39	<0.001^#^
Diastolic blood pressure, mmHg	78.89±6.26	80.87±6.53	81.17±7.02	0.001^#^
Total cholesterol, mmol/L	5.04 (4.37–5.61)	5.04 (4.46–5.85)	5.57 (4.93–6.29)	<0.001^*^
LDL cholesterol, mmol/L	3.21 (2.63–3.82)	3.21 (2.67–3.97)	3.59 (3.14–4.23)	<0.001^*^
HDL cholesterol, mmol/L	1.23 (1.08–1.40)	1.22 (1.09–1.43)	1.22 (1.08–1.45)	0.588^*^
Triglycerides, mmol/L	1.12 (0.85–1.63)	1.09 (0.80–1.55)	1.37 (0.97–1.96)	0.001^*^

## Discussion

Our study underlined the significant prevalence of traditional cardiovascular risk factors for coronary artery disease in the military population that increased with ageing. Furthermore, fibrinogen as a novel risk factor also grew with increasing age. Further analysis registered a positive correlation between fibrinogen and traditional risk factors values, but only ageing had a positive predictive value. Additional sub-analysis on the patients divided into terciles according to the fibrinogen values support previously cited results. Those finding seems to be very important, having in mind recent data considering age-related cardiovascular disease so-called« inflamm-ageing« [19]. This chronic low-grade inflammation state, pathophysiologically based on the agerelated increased inflammatory tone (inflamm-ageing) and nutrient excess (metaflammation) attributed to the accelerating vascular ageing and atherosclerosis per se. Except for accelerated atherosclerosis, there are also reciprocal positive interactions with traditional CV risk factors. All those findings contribute to creating novel therapeutic approaches that should promote healthy ageing and preserve health care system resources [20].

In order to define the preventive strategy goals, we analysed the prevalence of traditional cardiovascular risk factors among our specific study population. Most of our study group consisted of males younger than 40 with prehypertension in almost 75% and hypertension in 13.8% of participants. Among Serbian Armed Forces (older than 20 years of age), a significantly higher prevalence of prehypertension was identified than in the general population of the same age in the Republic of Serbia (in the age between 20 and 39 years, 67.4-54.1%; in the age between 40 and 44 years, 46.6%) [21] [22]. The prevalence of hypertension in the adult population of Serbia (aged ≥ 15 years) of 33.2% is significantly higher compared to hypertension among individuals in our study group (13.8%) [22].

Compared to results from the USA, the prevalence of hypertension in Serbia among the military population is more than 2.5 times higher. The proportion of hypertensive patients also increased with age; in the youngest group, there were only 6.4% of patients with hypertension, and in the oldest group, 21.4%, probably due to the so-called »lifestyle« that is characteristic for the group of uppermiddle-income countries, as Serbia [21] [22] [23] [24] [25]. Every candidate must go through a specific general medical examination and selection to become active military personnel. That may be the reason for the generally lower prevalence of hypertension in military personnel compared to the civilian population.

Besides blood pressure, both body mass and BMI index gradually increased with the age of the patients, so that the highest values were in the oldest group. Thus, our results are in accordance with the previous study considering trend, but the prevalence is a little higher, probably thanks to the specific nutrition habits and sedentary way of life among our study group [26] [27].

The average Serbian solder is, at least, overweight with a non-favourable LDL trend. American Heart Association (AHA) data underline that 36% of adults and 10% of children between 9 and 12 years have elevated cholesterol [17]. It seems important to consider that cumulative young adult exposures to elevated systolic BP, diastolic BP and LDL were associated with increased CVD risks in later life, independent of later adult exposures [28] [29]. Framingham study reported that males with total cholesterol over 8 mmol/L and females over the 6 mmol/L have almost 5 times higher risk for CVD in the next five years than the general population [18]. Recent data support the previous findings that normalisation of LDL cholesterol levels may lead to almost 40% CVD morbidity and mortality risk reduction [30] [31].

In 2006, according to the survey of the Ministry of Health of the Republic of Serbia, HLP incidence was 2.7% for males and 4.2% for females, and prevalence was 7.3% for males and 8.6% for females [18].

Our study pointed out that the prevalence of HLP increased with age, even in the so-called »healthy Warrior« population [13]. The prevalence of dyslipidemia among military personnel in the literature was from 5.3% to 41.96%. The prevalence of hypercholesterolemia, hypertriglyceridemia and low HDL-C are respectively: between 3.12% and 5.2%, 3.9% and 28%, 31% [14] [15] [21] [26] [27] [31]. In accordance with cited studies were our results considering all lipid fractions gradual increments with age.

Except traditional, we also analysed plasma fibrinogen levels as a novel cardiovascular risk factor for age-related cardiovascular disease and inflamm-ageing. Study data suggest that fibrinogen levels increase with ageing [32] [33]. There is a clear link between elevated plasma fibrinogen, cardiovascular disease and arterial and venous thrombosis [32]. The Framingham study confirmed a positive correlation between fibrinogen levels and risk of cardiovascular disease, as well as with the incidence of death and/or myocardial infarction [33]. Hyperfibrinogenemia is also an independent predictor of carotid thrombosis [34] [35]. The difference in plasma fibrinogen levels among hypertensive and normotensive patients was also registered [34]. It may be important, bearing in mind that among our study population older than 20, a higher prevalence of prehypertension than the general population of the same age in the Republic of Serbia was found [21] [22].

Our study analysis underlined ageing as an independent predictor influenced by the variability of fibrinogen. Fibrinogen levels were associated with traditional cardiovascular risk factors (blood pressure, total cholesterol, LDL cholesterol and triglycerides) and may not be influenced as much by body mass as CRP, supporting its usefulness as a biomarker of CVD [36]. Recent meta-analysis pointed out clear associations between fibrinogen level and the risks of CHD, stroke, other vascular and nonvascular mortality in healthy middle-aged adults [37]. Keeping in mind that fibrinogen levels predicted cardiovascular events independent of traditional risk factors in adults without clinical evidence of coronary heart disease at baseline, those findings may contribute to planning the strategy in primary prevention for these categories of individuals [38].

## Conclusions

Our results reported that military personnel with elevated blood pressure and dyslipidemias, followed by hyperfibrinogenemia, have multiple cardiovascular and cerebrovascular disease risk factors. Higher fibrinogen level is associated with traditional cardiovascular risk factors in this population and may be a useful biomarker of CVD in this high-risk subgroup. Those findings considering cardio and cerebrovascular risk factors would help create a new approach for primary prevention for these categories of individuals.

## Dodatak

### Author declaration

Authors certify that the manuscript represents a valid workpiece. Neither this manuscript nor one with substantially similar content under named authorship has been published or is being considered for publication elsewhere. The authors have participated in the research and the shaping of the manuscript.

### Acknowledgements

We thank the Ministry of Defense of the Republic of Serbia for their assistance and authorisation of the project Primary prevention of ischemic heart disease among active military personnel and civilian personnel in the military in the Republic of Serbia, MFVMA/5/17-19.

### Conflict of interest statement

The authors reported no conflict of interest regarding the publication of this article.
